# Common polymorphisms within the *NR4A3 *locus, encoding the orphan nuclear receptor Nor-1, are associated with enhanced β-cell function in non-diabetic subjects

**DOI:** 10.1186/1471-2350-10-77

**Published:** 2009-08-14

**Authors:** Peter Weyrich, Harald Staiger, Alena Stančáková, Silke A Schäfer, Kerstin Kirchhoff, Susanne Ullrich, Felicia Ranta, Baptist Gallwitz, Norbert Stefan, Fausto Machicao, Johanna Kuusisto, Markku Laakso, Andreas Fritsche, Hans-Ulrich Häring

**Affiliations:** 1 Department of Internal Medicine, Division of Endocrinology, Diabetology, Angiology, Nephrology, and Clinical Chemistry, University Hospital Tübingen, Tübingen, Germany; 2 Department of Medicine, Kuopio University Hospital, Kuopio, Finland

## Abstract

**Background:**

Neuron-derived orphan receptor (Nor) 1, nuclear receptor (Nur) 77, and nuclear receptor-related protein (Nurr) 1 constitute the NR4A family of orphan nuclear receptors which were recently found to modulate hepatic glucose production, insulin signalling in adipocytes, and oxidative metabolism in skeletal muscle. In this study, we assessed whether common genetic variation within the *NR4A3 *locus, encoding Nor-1, contributes to the development of prediabetic phenotypes, such as glucose intolerance, insulin resistance, or β-cell dysfunction.

**Methods:**

We genotyped 1495 non-diabetic subjects from Southern Germany for the five tagging single nucleotide polymorphisms (SNPs) rs7047636, rs1526267, rs2416879, rs12686676, and rs10819699 (minor allele frequencies ≥ 0.05) covering 100% of genetic variation within the *NR4A3 *locus (with D' = 1.0, r^2 ^≥ 0.9) and assessed their association with metabolic data derived from the fasting state, an oral glucose tolerance test (OGTT), and a hyperinsulinemic-euglycemic clamp (subgroup, N = 506). SNPs that revealed consistent associations with prediabetic phenotypes were subsequently genotyped in a second cohort (METSIM Study; Finland; N = 5265) for replication.

**Results:**

All five SNPs were in Hardy-Weinberg equilibrium (p ≥ 0.7, all). The minor alleles of three SNPs, i.e., rs1526267, rs12686676, and rs10819699, consistently tended to associate with higher insulin release as derived from plasma insulin at 30 min(OGTT), AUC_C-peptide_-to-AUC_Gluc _ratio and the AUC_Ins30_-to-AUC_Gluc30 _ratio with rs12686676 reaching the level of significance (p ≤ 0.03, all; additive model). The association of the SNP rs12686676 with insulin secretion was replicated in the METSIM cohort (p ≤ 0.03, additive model). There was no consistent association with glucose tolerance or insulin resistance in both study cohorts.

**Conclusion:**

We conclude that common genetic variation within the *NR4A3 *locus determines insulin secretion. Thus, *NR4A3 *represents a novel candidate gene for β-cell function which was not covered by the SNP arrays of recent genome-wide association studies for type 2 diabetes mellitus.

## Background

The NR4A family of orphan nuclear receptors comprises nuclear receptor (Nur) 77 (gene: *NR4A1*), nuclear receptor-related protein (Nurr) 1 (gene: *NR4A2*), and neuron-derived orphan receptor (Nor) 1 (gene: *NR4A3*). These ligand-independent constitutively active transcription factors are co-expressed in many metabolically relevant tissues, such as skeletal muscle, adipose tissue, liver, heart, and brain, and are thought to be predominantly regulated at the transcriptional level (for review, see [1]). In this context, a plethora of stimuli was identified, including fatty acids [2], growth factors [3], inflammatory cytokines [4,5], peptide hormones [6,7], membrane depolarisation [8], and stress [9], regulating NR4A family member expression in a tissue-specific manner. Recently, important metabolic functions of NR4A transcription factors were revealed. All three NR4A family members are induced in the liver upon fasting and glucagon stimulation, and their hepatic expression was found to be increased in both type 1 and type 2 diabetic mouse models [10].

Moreover, adenoviral over-expression of these receptors induced the expression of gluconeogenetic enzymes and enhanced hepatic glucose production in vitro as well as in vivo [10]. In 3T3-L1 adipocytes, *NR4A1 *(Nur-77) and *NR4A3 *(Nor-1) expression was reported to be induced upon insulin and thiazolidinedione treatment.

Furthermore, the expression of both receptors was found to be decreased in skeletal muscle and adipose tissue of multiple rodent models of insulin resistance and diabetes [11]. As lentiviral Nor-1 over-expression additionally was shown to enhance adipocyte insulin signalling and glucose transporter-4 translocation [11] and siRNA knock-down showed that Nor-1 regulates gene expression for oxidative metabolism in skeletal muscle [12], we asked whether common genetic variation within the genetic locus harbouring the Nor-1 gene *NR4A3 *(OMIM ID 600542, Entrez Gene ID 8013) contributes to the development of prediabetic phenotypes, especially such as glucose intolerance and insulin resistance.

To this end, we genotyped 1495 non-diabetic subjects from Southern Germany for the five tagging single nucleotide polymorphisms (SNPs) rs7047636, rs1526267, rs2416879, rs12686676, and rs10819699 (minor allele frequencies (MAFs) ≥ 0.05) covering 100% of genetic variation within the *NR4A3 *locus (with D' = 1.0 and r^2 ^≥ 0.9) and assessed their association with metabolic data derived from the fasting state, an oral glucose tolerance test (OGTT), and, in a subgroup of 506 subjects, from a hyperinsulinemic-euglycemic clamp. Found SNP-genotype associations were subjected to further analysis in the METSIM Study cohort from Finland (N = 5265) for replication purposes.

## Methods

### Study participants

#### TÜF/TULIP cohort

Data from 1495 non-diabetic participants of the **Tü**bingen **F**amily Study and the **Tu**ebingen **L**ifestyle **I**ntervention **P**rogram [13] were analyzed for this study. The participants (characteristics given in Table 1) did not take any medication known to affect glucose tolerance or insulin secretion, and were requested not to smoke 24 h before and during the test period. Informed written consent was obtained from all study participants, and the local ethics committee has approved the protocol.

**Table 1:** Clinical characteristics of the TÜF/TULIP and METSIM study population.

	**TÜF/TULIP**	**METSIM**
Gender (female/male)	989/506	0/5265
IFG/IGT/(IFG+IGT)	150/142/113	874/498/343
Age (y)	39 ± 13	58 ± 6
BMI (kg/m^2^)	28.6 ± 8.0	26.8 ± 3.8
Body Fat (%)	30.6 ± 10.5	23.9 ± 6.5
Fasting glucose (mM)	5.10 ± 0.55	5.69 ± 0.50
Glucose, 120 min OGTT (mM)	6.24 ± 1.66	6.09 ± 1.69
Fasting insulin (pM)	62.4 ± 51.2	48.6 ± 33.8
Insulin, 30 min OGTT (pM)	481 ± 384	391 ± 284

Data are given as means ± SD. BMI – body mass index; IFG – impaired fasting glucose (defined as 100–126 mg/dl according to AHA guidelines); IGT – impaired glucose tolerance; OGTT – oral glucose tolerance test.

#### METSIM cohort

The **MET**abolic **S**yndrome **I**n **M**en (METSIM) Study addresses a random sample of 6147 Finnish men aged from 45 to 70 years in Eastern Finland (Kuopio, see Table 1). All study participants underwent an oral glucose tolerance test (OGTT, see below), and the study protocol was approved by the local ethics committee. For the study of prediabetic traits, only non-diabetic METSIM participants were analyzed (N = 5265, aged 58 ± 6 years). METSIM participants with newly diagnosed diabetes (N = 882) according to the WHO criteria [14] allowed estimation for diabetes risk using logistic regression analysis.

### Anthropometrics

Body mass index (BMI) was calculated as weight divided by the square of height (kg/m^2^). Body fat was determined by bioelectrical impedance (RJL, Detroit, MI, USA).

### Analytical procedures

Blood glucose was measured with a bedside glucose analyzer (Yellow Springs Instruments, Yellow Springs, OH, USA). Plasma insulin and C-peptide concentrations were measured by commercial chemiluminescence assays for ADVIA Centaur (Siemens Medical Solutions, Fernwald, Germany) according to the manufacturer's instructions. In the METSIM Study, plasma glucose was measured by enzymatic hexokinase photometric assay (Konelab Systems Reagents, Thermo Fischer Scientific, Vantaa, Finland), and insulin was determined by immunoassay (ADVIA Centaur Insulin IRI, no 02230141, Siemens Medical Solutions Diagnostics, Tarrytown, NY).

### Oral glucose tolerance test

The oral glucose tolerance test was performed according to the WHO recommendations after a 12-h fasting period [14]. In addition to plasma glucose, C-peptide levels and plasma insulin were measured at 0, 30, 60, 90 and 120 min in the TÜF/TULIP cohort. In the METSIM Study, plasma glucose and insulin levels were measured at 0, 30 and 120 min.

### Calculations on insulin sensitivity and insulin secretion

Insulin sensitivity was estimated from insulin and glucose values obtained during the OGTT according to the (HOMA) homeostasis model assessment [15] or the method proposed by Matsuda and DeFronzo [16]. In 506 TÜF/TULIP Study participants, insulin sensitivity was additionally measured by a euglycemic hyperinsulinemic clamp, as described elsewhere [17]. In TÜF/TULIP, insulin secretion was estimated from insulin plasma levels measured at 30 min of the OGTT and the area under the curve (AUC) of C-peptide levels divided by the corresponding AUC of plasma glucose (AUC_C-peptide_-to-AUC_Gluc _ratio) levels [18]. In both study cohorts, insulin secretion was estimated from insulin plasma levels measured at 30 min during the OGTT and from the AUC_Ins30_-to-AUC_Gluc30 _ratio [(insulin_0 min _+ insulin_30 min_)/glucose_0 min _+ glucose_30 min_)] that recently has been shown to be the best surrogate parameter for first phase insulin secretion in an intravenous glucose tolerance test [19].

### Genotyping and selection of tagging SNPs

Genomic DNA was isolated from EDTA blood samples by blood cell lysis, protein precipitation and a washing protocol as previously described [17]. Genotyping was accomplished by use of the TaqMan Assay (Eurogentec, Liege, Belgium) and an ABI Prism 7500 sequence detection system (Applied Biosystems, Foster City, CA, USA) for selected tagging SNPs. Selection criteria for tagging SNPs of the *NR4A3 *locus on chromosome 9q22 were a minor allele frequency (MAF) ≥ 0.05 and a linkage disequilibrium measure r^2 ^≤ 0.8 in the CEU population of the HapMap project (Utah residents with ancestry from Northern and Western Europe).

### Gene expression analyses in human islet and adipose tissue

Pancreata were obtained from brain-dead multiorgan donors and islet isolations were performed as previously described according to the Ricordi method with local (Geneva) adaptations [20,21]. Briefly, the pancreas was distended by intraductal infusion of a cold collagenase solution. After digestion at 37°C in a modified Ricordi chamber, separated exocrine and endocrine tissues were washed and purified in a continuous Biocoll gradient. After several washings, islets were incubated in CMRL 1066 medium containing 5.6 mM glucose. RNA was extracted from cultivated (2d) human islets and adipose tissue specimens with PeqGOLD Tri Fast™ (PEQLAB Biotechnologie, Erlangen, Germany) according to the protocol supplied by the manufacturer, and quantitative real-time PCR was performed with Roche's LightCycler^® ^480 (Roche, Basel, Switzerland).

### Statistical analyses

The Hardy-Weinberg equilibrium was tested with the χ^2^-test. Three-group comparisons without adjustments were performed using ANOVA. In addition, multivariate linear regression models with adjustments to relevant covariates were undertaken for genotype-phenotype association analyses. Non-normally distributed parameters were log-transformed. All data are presented as means ± SD. The software package JMP 7.0 (SAS Institute, Cary, NC, USA) and SPSS 14.0 (SPSS, Chicago, IL, USA) was used for statistical analysis. A *p*-value < 0.05 was considered to be statistically significant. Linkage disequilibrium was analyzed with the JLIN software [22].

## Results

### Study population

The TÜF/TULIP Study cohort of 1495 (506 male/989 female) non-diabetic subjects had a mean age of 39 ± 13 years. 10% had an impaired fasting glucose (IFG), 9.5% showed an impaired glucose tolerance (IGT) and 7.6% presented with both IFG and IGT in the OGTT. METSIM non-diabetic participants (N = 5265 men) had an average age of 58 ± 6 years, and 16.6 (9.5; 6.5)% were diagnosed with IFG (IGT; IFG+IGT; see Table 1). A total of 882 METSIM participants (59 ± 6 years) genotyped for *NR4A3 *were diagnosed with diabetes. Further details on basal anthropometric and metabolic traits of the investigated populations are provided in Table 1.

### Genetic analyses

**Figure 1:**
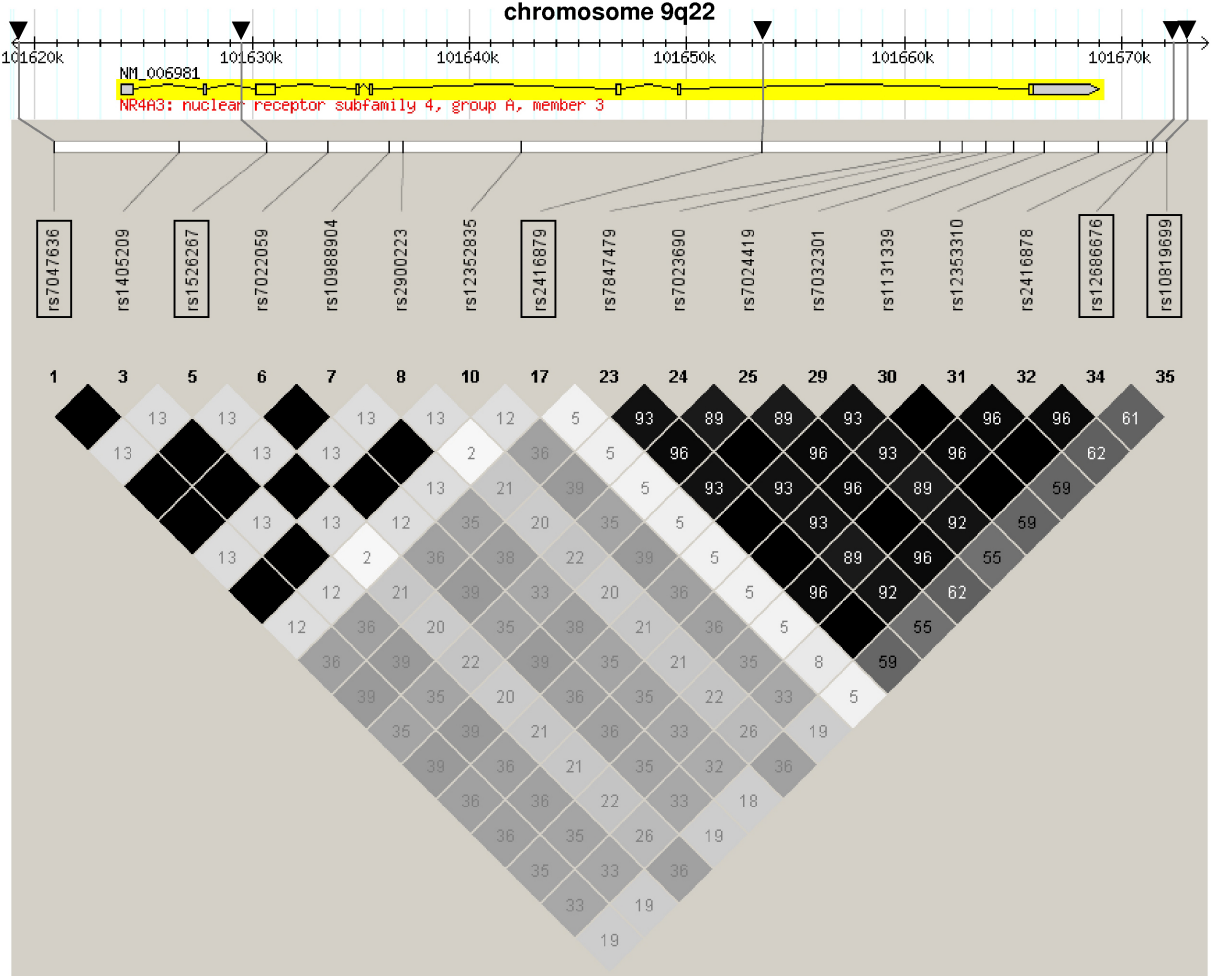
**Fifty-five-kb genomic region of human chromosome 9q22 harbouring the NR4A3 gene and HapMap LD data of the 17 common (minor allele frequency ≥ 0.05) informative SNPs within this region**. The *NR4A3 *gene consists of eight exons and seven introns and spans 45 kb from nucleotide 101,623,958 to nucleotide 101,668,994. The locations of the five tagging SNPs rs7047636, rs1526267, rs2416879, rs12686676, and rs10819699 are indicated by black arrows. The Haploview LD colour scheme 'R-squared' was chosen to visualize regions of high linkage disequilibrium (black diamonds: complete LD; dark grey diamonds: strong LD; light grey diamonds: weak LD). Within the diamonds, the r^2 ^values are given (black diamonds: r^2 ^= 1.0). LD – linkage disequilibrium; SNP – single nucleotide polymorphism.

HapMap analysis of the *NR4A3 *locus revealed five tagging SNPs (rs7047636, rs1526267, rs2416879, rs12686676 and rs10819699) to cover 100% of variation in the *NR4A3 *gene locus (55 kb) including 5 kb of its 5'-flanking region and 5 kb of its 3'-flanking region (see Figure 1). Genotype call rates were > 98.4% for all investigated SNPs, and minor allele frequencies of the tagging SNPs ranged from 0.10 (rs2416879) to 0.46 (rs12686676; see Table 2) in the TÜF/TULIP cohort. All SNPs were in Hardy-Weinberg equilibrium (p ≥ 0.7, all; χ^2^-test), and linkage disequilibrium of the five selected tagging SNPs was rather low (r^2 ^< 0.6, all; see Table 2).

**Table 2:** Linkage disequilibrium statistics (D', r^2^) among the five tagging SNPs rs7047636, rs1526267, rs2416879, rs12686676, and rs10819699 covering the 55-kb genomic locus harbouring the NR4A3 gene in the screening population (TÜF/TULIP cohort).

SNP	**rs7047636**	**rs1526267**	**rs2416879**	**rs12686676**	**rs10819699**
MAF	0.371	0.256	0.100	0.464	0.319
rs7047636	-	1.000	1.000	0.892	0.903
rs1526267	0.202	-	1.000	0.767	0.793
rs2416879	0.065	0.038	-	0.975	0.952
rs12686676	0.406	0.233	0.091	-	1.000
rs10819699	0.225	0.461	0.047	0.540	-

D' values above empty cells; r² values below empty cells. SNP – single nucleotide polymorphism, MAF – minor allele frequency

### Association of *NR4A3 *SNPs with anthropometric and metabolic traits in TÜF/TULIP

We found a significant association of the minor allele of rs7047636 with increased plasma glucose at 120 min of the OGTT (p = 0.03, additive model, adjusted for age, gender and BMI). The minor allele of rs1526267 was associated with a higher BMI (p = 0.02, additive model, adjusted for age and gender) and with decreased insulin sensitivity measured by clamp (p = 0.03, additive model, adjusted for age, gender and BMI). We did not detect any significant association of the four other tagging SNPs with BMI, body fat, blood glucose levels (fasting state and 120 min OGTT) and insulin sensitivity (see Table 3 and [Additional file 1]). The most consistent finding in the TÜF/TULIP population was a trend towards an association of the minor alleles of rs1526267, rs12686676 and rs10819699 with increased insulin secretion, with rs12686676 reaching the level of significance (p ≤ 0.03 after adjustment for age, gender, BMI and insulin sensitivity; Table 3). This finding was independent of the insulin secretion estimation model used for the analysis (insulin at 30 min OGTT, AUC_C-peptide_-to-AUC_Gluc _ratio, AUC_Ins30_-to-AUC_Gluc30 _ratio; Table 3). All these findings could be reproduced if the Matsuda model [16] instead of the HOMA model [15] was used as an estimate of insulin sensitivity. In the dominant model of e.g. AUC_C-peptide_-to-AUC_Gluc _ratio, the increase in insulin secretion amounted to 6.2% for rs1526267, 6.0% for rs12686676 and 3.7% for rs10819699. The intronic SNP rs1526267 is in a very low LD with the *NR4A3*-3'-flanking SNPs rs12686676 and rs10819699 in both the CEU population of the HapMap project (Figure 1) and our cohort (Table 2), and the fact that three rather independent SNPs associate with insulin secretion further underscores the role of *NR4A3 *in beta-cell function. The SNP rs2416879 also showed a significant association with two insulin secretion estimation models (insulin at 30 min OGTT, AUC_Ins30_-to-AUC_Gluc30 _ratio; p < 0.001 for adjusted models, [see Additional file 1]). However, no clear allele dose effect could be detected. Therefore, we doubted about the reliability of this finding and did not replicate this SNP in the METSIM Study.

**Table 3:** Associations of *NR4A3 *SNPs rs1526267, rs12686676 and rs10819699 with anthropometric and metabolic data (TÜF/TULIP cohort, N = 1495)

SNP	rs1526267					
				
Genotype	GG	GA	AA	p_1_	p_2_	p_3_
N	832	561	102	-	-	-
Age (y)	39 ± 13	39 ± 13	38 ± 14	0.9	0.9	-
BMI (kg/m^2^)	28.0 ± 7.2	29.4 ± 8.9	28.5 ± 8.4	**0.0234**	**0.0225**	-
Body Fat (%)	30.5 ± 10	31.0 ± 11	29.8 ± 10	0.7	0.5	-
Fasting glucose (mM)	5.09 ± 0.54	5.11 ± 0.56	5.07 ± 0.55	0.8	0.9	-
Glucose, 120 min OGTT (mM)	6.25 ± 1.65	6.26 ± 1.68	6.01 ± 1.56	0.4	0.3	-
Insulin sensitivity (HOMA), OGTT (AU)	2.28 ± 2.02	2.61 ± 2.34	2.50 ± 2.08	**0.0186**	0.17	-
Insulin sensitivity, clamp (U)*	0.090 ± 0.059	0.079 ± 0.047	0.085 ± 0.054	**0.0549**	**0.0274**	-
AUC_C-peptide_-to-AUC_Gluc _ratio, OGTT (·10^-9^)	310 ± 100	329 ± 114	331 ± 115	**0.0054**	**0.0241**	0.07
AUC_Ins30_-to-AUC_Gluc30 _ratio, OGTT (·10^-9^)	38.3 ± 28	43.3 ± 33	42.5 ± 31	**0.0055**	0.08	0.3
Insulin, 30 min OGTT (pM)	393 ± 266	501 ± 373	459 ± 372	**0.0020**	**0.0443**	0.13
SNP	rs12686676					
				
Genotype	GG	GA	AA	p_1_	p_2_	p_3_
N	424	754	317	-	-	-
Age (y)	38 ± 13	39 ± 13	39 ± 13	0.3	0.2	-
BMI (kg/m^2^)	28.0 ± 7.7	28.8 ± 8.0	28.7 ± 8.2	0.1	0.2	-
Body Fat (%)	30.0 ± 11	31.1 ± 10	30.4 ± 11	0.1	0.3	-
Fasting glucose (mM)	5.09 ± 0.54	5.11 ± 0.56	5.07 ± 0.55	0.7	0.6	-
Glucose, 120 min OGTT (mM)	6.23 ± 1.66	6.32 ± 1.63	6.06 ± 1.70	**0.0335**	0.06	-
Insulin sensitivity (HOMA), OGTT (AU)	2.28 ± 1.97	2.53 ± 2.32	2.35 ± 1.95	0.14	0.24	-
Insulin sensitivity, clamp (U)*	0.091 ± 0.065	0.083 ± 0.050	0.085 ± 0.050	0.7	0.4	-
AUC_C-peptide_-to-AUC_Gluc _ratio, OGTT (·10^-9^)	306 ± 107	325 ± 107	320 ± 105	**0.0055**	**0.0142**	**0.028**
AUC_Ins30_-to-AUC_Gluc30 _ratio, OGTT (·10^-9^)	37.9 ± 29	41.7 ± 31	40.9 ± 29	**0.0041**	**0.010**	**0.020**
Insulin, 30 min OGTT (pM)	388 ± 301	445 ± 309	478 ± 357	**0.0017**	**0.0058**	**0.012**

SNP	rs10819699					
				
Genotype	GG	GA	AA	p_1_	p_2_	p_3_
N	698	637	158	-	-	-
Age (y)	39 ± 13	39 ± 13	38 ± 14	0.5	0.5	-
BMI (kg/m^2^)	28.3 ± 7.6	28.8 ± 8.1	29.0 ± 8.9	0.4	0.5	-
Body Fat (%)	30.4 ± 10	31.1 ± 10	29.8 ± 11	0.3	0.7	-
Fasting glucose (mM)	5.09 ± 0.55	5.10 ± 0.55	5.09 ± 0.56	1.0	0.9	-
Glucose, 120 min OGTT (mM)	6.23 ± 1.63	6.29 ± 1.67	6.08 ± 1.68	0.3	0.5	-
Insulin sensitivity (HOMA), OGTT (AU)	2.38 ± 2.27	2.48 ± 2.08	2.39 ± 1.91	0.68	0.24	-
Insulin sensitivity, clamp (U)*	0.087 ± 0.058	0.084 ± 0.053	0.087 ± 0.050	0.8	0.2	-
AUC_C-peptide_-to-AUC_Gluc _ratio, OGTT (·10^-9^)	313 ± 105	324 ± 110	325 ± 103	0.07	0.1	0.2
AUC_Ins30_-to-AUC_Gluc30 _ratio, OGTT (·10^-9^)	38.9 ± 30	41.9 ± 31	41.3 ± 29	**0.0349**	**0.0538**	0.18
Insulin, 30 min OGTT (pM)	396 ± 288	471 ± 339	372 ± 352	**0.0125**	**0.0201**	0.08

Data are given as means ± SD. For statistical analysis, data were log-transformed. **p_1 _**– non-adjusted additive model; **p_2 _**– adjusted additive model: age was adjusted for gender; BMI and body fat were adjusted for gender and age; glucose (Gluc) levels as well as insulin sensitivity (HOMA model, clamp) and insulin (Ins) secretion (AUC_C-peptide_-to-AUC_Gluc _ratio, AUC_Ins30_-to-AUC_Gluc30 _ratio, plasma insulin at 30 min OGTT) were adjusted for gender, age, and BMI; **p_3 _**– Ins secretion parameters with adjustment for age, BMI, gender and insulin sensitivity. *clamped subgroup (N = 506). AUC – area under the curve; BMI – body mass index; OGTT – oral glucose tolerance test; SNP – single nucleotide polymorphism.

### Replication in the METSIM Study

Next, we tried to replicate the most reliable associations between SNPs (rs1526267, rs12686676, rs10819699) and insulin secretion in the METSIM cohort. Adjustment for age, BMI and insulin sensitivity (HOMA) revealed a significant association with both insulin secretion estimation models (insulin at 30 min OGTT, AUC_Ins30_-to-AUC_Gluc30 _ratio) in rs12686676, which yet showed the most convincing association in the TÜF/TULIP Study. In contrast to TÜF/TULIP, rs1526267 had no effect on BMI or insulin sensitivity in METSIM participants, and both rs1526267 and rs10819699 did not associate with insulin secretion (Table 4). Since the METSIM Study design also allows the analysis of *NR4A3 *genetic variants for the endpoint diabetes in a population-based cohort, we tested whether rs1526267, rs12686676 or rs10819699 associated with the prevalence of diabetes. However, comparing diabetic with non-diabetic study participants by logistic regression analysis did not reveal a significantly altered diabetes prevalence in minor allele carriers of the three investigated SNPs (p > 0.11; all, [see Additional file 2]). There were also no significant differences in genotype distribution of the investigated *NR4A3 *SNPs according to the glucose tolerance status (normal glucose tolerance, impaired fasting glucose, impaired glucose tolerance, diabetes) of both TUEF/TULIP and METSIM participants [see Additional file 3].

**Table 4:** Associations of NR4A3 SNPs rs1526267, rs12686676 and rs10819699 with metabolic data and insulin secretion indices (METSIM cohort, N = 5265)

SNP	rs1526267					
				
Genotype	GG	GA	AA	p_1_	p_2_	p_3_
N	2311	2317	637	-	-	-
Age (y)	58 ± 6	58 ± 7	58 ± 6	0.3	-	-
BMI (kg/m^2^)	26.8 ± 3.7	26.8 ± 3.8	26.7 ± 3.7	0.5	-	-
Insulin sensitivity (HOMA), OGTT (AU)	2.10 ± 1.55	2.11 ± 1.54	1.95 ± 1.32	0.14	0.12	-
AUC_Ins30_-to-AUC_Gluc30 _ratio, OGTT (·10^-9^)	34.7 ± 24	35.9 ± 25	34.0 ± 24	**0.0381**	0.08	0.12
Insulin, 30 min OGTT (pM)	445 ± 320	464 ± 340	437 ± 314	**0.040**	0.10	0.10
SNP	rs12686676					
				
Genotype	GG	GA	AA	p_1_	p_2_	P_3_
N	1127	2520	1585	-	-	-
Age (y)	58 ± 6	59 ± 7	58 ± 6	0.3	-	-
BMI (kg/m^2^)	26.8 ± 3.6	26.9 ± 3.9	26.7 ± 3.7	0.14	-	-
Insulin sensitivity (HOMA), OGTT (AU)	2.02 ± 1.40	2.12 ± 1.58	2.10 ± 1.54	0.3	0.6	-
AUC_Ins30_-to-AUC_Gluc30 _ratio, OGTT (·10^-9^)	34.3 ± 23	36.2 ± 25	34.4 ± 23	**0.021**	0.09	**0.029**
Insulin, 30 min OGTT (pM)	441 ± 320	466 ± 345	442 ± 314	**0.025**	0.11	**0.038**

SNP	rs10819699					
				
Genotype	GG	GA	AA	p_1_	p_2_	p_3_
N	1830	2480	913	-	-	-
Age (y)	58 ± 6	58 ± 7	58 ± 6	0.6	-	-
BMI (kg/m^2^)	26.8 ± 3.7	26.9 ± 3.8	26.8 ± 3.8	0.5	-	-
Insulin sensitivity (HOMA), OGTT (AU)	2.11 ± 1.61	2.09 ± 1.47	2.02 ± 1.47	0.3	0.3	-
AUC_Ins30_-to-AUC_Gluc30 _ratio, OGTT (·10^-9^)	34.9 ± 24	35.6 ± 24	35.1 ± 25	0.2	0.5	0.4
Insulin, 30 min OGTT (pM)	447 ± 325	459 ± 332	451 ± 336	0.2	0.4	0.3

Data are given as means ± SD. For statistical analysis, data were log-transformed. **p_1 _**– non-adjusted additive model; **p_2 _**– additive model with adjustements to BMI and age; **p_3 _**– AUC_Ins30_-to-AUC_Gluc30 _ratio and plasma insulin at 30 min OGTT with adjustements for age, BMI and insulin sensitivity; AUC – area under the curve; OGTT – oral glucose tolerance test; SNP – single nucleotide polymorphism, Gluc – Glucose, Ins – Insulin.

### NR4A3 expression in human islets

**Figure 2:**
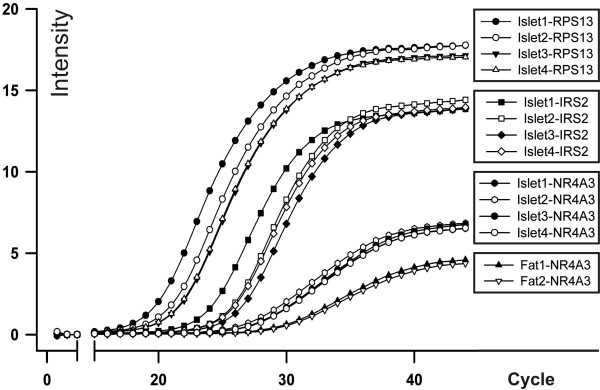
***NR4A3 *gene expression in human islets**. RNA was isolated from four independent (islet1-4) freshly isolated human islet preparations, and NR4A3 gene expression was determined with quantitative real-time PCR in comparison with the house-keeping gene RPS13 (40S ribosomal protein S13) and insulin-receptor-substrate 2 (IRS2). RNA preparations of two adipose tissue samples were included as a positive control for NR4A3 expression analysis.

NR4A3 gene expression was determined in four independent, freshly isolated, human islet preparations. Signal intensities of NR4A3 were comparable for all four islet preparations. Interestingly, NR4A3 signals were ~1.5 fold stronger in human islets as in two samples of adipose tissue included as a positive control, and remarkably high in comparison to two other positive controls, namely the house-keeping gene *RPS13 *(40S ribosomal protein S13) and *IRS2 *(insulin-receptor-substrate 2; Figure 2).

## Discussion

This study investigated the role of *NR4A3 *for metabolic traits in a prediabetic population. Our primary hypothesis was that *NR4A3 *variants may associate with glucose intolerance or insulin resistance, based on published data on Nor-1 functions in liver and skeletal muscle [10-12,23]. However, we did not detect any convincing associations between the selected *NR4A3 *tagging SNPs and glucose intolerance/insulin resistance in the two investigated study cohorts. Contrary to our expectation, the principal finding was that three out of the five investigated tagging SNPs, namely rs1526267, rs12686676, and rs10819699, showed a consistent trend towards an association with insulin secretion in the TÜF/TULIP cohort, with rs12686676 reaching a significant level after adjustment for confounding variables. This finding could be replicated in a second independent cohort of Finnish male subjects (METSIM Study). Summarizing our data obtained from both cohorts, we assume a rather dominant effect of *NR4A3 *on insulin secretion. However, further research is needed to verify this hypothesis in other cohorts, as all other detected diabetes risk genes show an additive allele effect [24]. It is of note that the tested SNPs were not covered by the SNP arrays used in recent genome-wide association studies (GWA), according to currently available statistic data . Interestingly, most diabetes genes detected by the GWA studies are also involved in insulin secretory function [25-28], as it is the case for *NR4A3*.

As the effect of *NR4A3 *on insulin secretion was not dependent on the levels of non-esterified fatty acids as a read-out of adipose tissue metabolism determined in our study cohort (data not shown), we assume that *NR4A3 *genetic variability is an independent determinant for insulin secretion. Our finding of a remarkably high *NR4A3 *gene expression in freshly isolated human islets also supports a physiological role of *NR4A3 *in beta cells. Further evidence for this hypothesis arises from a study on target genes of a fusion protein encoded by a t(9;22) chromosomal translocation. The resulting EWS/NOR-1 fusion induces serum- and glucocorticoid-regulated kinase 1 (SGK1) [29], which mediates inhibition of insulin release in insulin-secreting cells [30].

As mentioned above, there was no replicable significant effect of *NR4A3 *genetic variability on insulin sensitivity in the two study populations, despite that both *NR4A1 *and *NR4A3 *expression is reduced in skeletal muscle of various diabetic animal models [11] and increased in L6 skeletal muscle cells and muscle biopsies upon insulin stimulation [31] or dietary restriction [32]. We also were not able to show a replicable association with BMI in our two study cohorts. This finding may reflect the fact that the NR4A family of nuclear orphan receptors was shown to be dispensable for adipogenesis, at least in the 3T3L1 adipocyte cellular model [33].

The following limitations of our study should be addressed: we did not a priori correct our data for multiple comparisons. Bonferroni correction for 5 independently analyzed SNPs (corrected α-level: 0.0102) would render all reported findings nominal. However, association with insulin secretion was consistently seen in three tagging SNPs in the *NR4A3 *gene and independent of the model used for estimation of insulin secretion (plasma insulin at 30 min OGTT, AUC_C-peptide_-to-AUC_Gluc _ratio, AUC_Ins30_-to-AUC_Gluc30 _ratio) in the TÜF/TULIP Study cohort, and the most significant association of rs12686676 could be replicated in a second larger population (METSIM Study). As recent GWA studies mainly detected genes involved in insulin secretion as diabetes risk genes [24], one may assume that *NR4A3 *genetic variants have an impact on diabetes prevalence. Surprisingly, this was not the case in the METSIM cohort. As the METSIM study (diabetes prevalence: 13.8%) provides a power of 80% to detect a genotype relative risk of 1.15 assuming a SNP minor allele frequency of ~0.35 [see Additional file 3], we conclude that NR4A3 genetic variability may confer a rather modest effect on diabetes risk, and that analysis of this locus necessitates even larger replication samples.

## Conclusion

We are confident that the described association between *NR4A3 *genetic variants and insulin secretion is not a by-chance finding, and that further research on the role of orphan nuclear receptors in beta cell function and diabetes pathogenesis is warranted. In addition, it would be interesting to investigate the role of *NR4A3 *genetic variants in large studies employing a case-control design.

## Competing interests

The authors declare that they have no competing interests.

## Authors' contributions

PW and HS were responsible for the complete statistical analysis and prepared all tables, figures and the manuscript. AS did the complete statistical analysis for the METSIM cohort and prepared tables and the manuscript. SAS and KK made contributions to phenotyping of TÜF/TULIP participants. FM is responsible of the genotyping facility at the University of Tübingen and additionally performed the real-time PCR studies. SU, FR and BG provided freshly isolated human islet preparations. ML and JK are the principal investigators of the METSIM Study and responsible for METSIM patient and data management. NS, AF and HUH acted as principal investigators for the TÜF/TULIP study, and were responsible for patient and data management. All authors read and approved the final manuscript.

## Pre-publication history

The pre-publication history for this paper can be accessed here:



## Supplementary Material

Additional file 1 **Associations of *NR4A3 *SNPs rs7047636 and rs2416879 with anthropometric and metabolic data (TÜF/TULIP cohort, N = 1495)**. Data are given as means ± SD. For statistical analysis, data were log-transformed. p_1 _– non-adjusted additive model; p_2 _– adjusted additive model: age was adjusted for gender; BMI and body fat were adjusted for gender and age; glucose (Gluc) levels as well as insulin sensitivity (HOMA model, clamp) and insulin (Ins) secretion (AUC_C-peptide_-to-AUC_Gluc _ratio, AUC_Ins30_-to-AUC_Gluc30 _ratio, plasma insulin at 30 min OGTT) were adjusted for gender, age, and BMI; p_3 _– Ins secretion parameters with adjustment for age, BMI, gender and insulin sensitivity. *clamped subgroup (N = 506). AUC – area under the curve; BMI – body mass index; OGTT – oral glucose tolerance test; SNP – single nucleotide polymorphism. Click here for file

Additional file 2 **Logistic regression analysis for differences of NR4A3 SNP's minor allele prevalences in individuals with normal glucose tolerance (NGT) and overt diabetes mellitus (DM) in the METSIM Study**. The [95%] confidence interval of the data provided is indicated for all different statistical models (additive, dominant, recessive) for all investigated SNPs. Click here for file

Additional file 3 **Distribution of NR4A3 SNP minor allele frequencies according to glucose tolerance status in the TÜF/TULIP (N = 1495) and the METSIM (N = 6147) cohort**. Minor allele frequencies for each investigated NR4A3 SNP are presented for study participants (TUEF-TULIP/METSIM trial) with normal glucose tolerance (NGT), impaired fasting glucose and/or impaired glucose tolerance (IFG/IGT) and with manifest diabetes (METSIM participants only). p_1 _– NGT vs. IFG/IGT in TUEF-TULIP/METSIM(χ^2^-test); p_2 _– NGTvs.IFG/IGTvs.DIABETESinMETSIM(χ^2^-test). SNPs screened in TUEF/TULIP only and not replicated in METSIM are marked with an asterisk. Click here for file
