# Supplementary material for: Common polymorphisms within the NR4A3 locus, encoding the orphan nuclear receptor Nor-1, are associated with enhanced β-cell function in non-diabetic subjects
Source: BMC Med Genet. 2009 Aug 14;10:77. doi: 10.1186/1471-2350-10-77 (PMC2741445; doi:10.1186/1471-2350-10-77)
Supplement: Additional file 2 — Logistic regression analysis for differences of NR4A3 SNP's minor allele prevalences in individuals with normal glucose tolerance (NGT) and overt diabetes mellitus (DM) in the METSIM Study. The [95%] confidence interval of the data provided is indicated for all different statistical models (additive, dominant, recessive) for all investigated SNPs. [file 1471-2350-10-77-S2.doc]

**Additional file 2 –** Logistic regression analysis for differences of NR4A3 SNP’s minor allele prevalences in individuals with normal glucose tolerance (NGT) and overt diabetes mellitus (DM) in the METSIM Study.

| **NGT vs. DM unadjusted** | | |  |  |  |  |  | **NGT vs. DM adjusted for age and BMI** | | | |  |  |  |
| --- | --- | --- | --- | --- | --- | --- | --- | --- | --- | --- | --- | --- | --- | --- |
| SNP | Model |  | ExpB | Confid. interval | | Sig |  | SNP | Model |  | ExpB | Confid. interval | | Sig |
|  |  |  |  | Lower | Upper |  |  |  |  |  |  | Lower | Upper |  |
| rs1526267 | additive | CTvsCC | 1,045 | 0,893 | 1,223 | 0,581 |  | rs1526267 | additive | CTvsCC | 1,007 | 0,847 | 1,197 | 0,938 |
|  |  | TTvsCC | 0,988 | 0,774 | 1,261 | 0,922 |  |  |  | TTvsCC | 0,962 | 0,734 | 1,260 | 0,779 |
|  | dominant | CCvsCT+TT | 1,033 | 0,890 | 1,198 | 0,669 |  |  | dominant | CCvsCT+TT | 0,997 | 0,847 | 1,175 | 0,976 |
|  | recessive | CC+CTvsTT | 0,966 | 0,767 | 1,217 | 0,772 |  |  | recessive | CC+CTvsTT | 0,959 | 0,743 | 1,238 | 0,747 |
| rs12686676 | additive | AGvsAA | 1,020 | 0,859 | 1,211 | 0,824 |  | rs12686676 | additive | AGvsAA | 0,987 | 0,816 | 1,193 | 0,892 |
|  |  | GGvsAA | 1,063 | 0,866 | 1,305 | 0,561 |  |  |  | GGvsAA | 1,162 | 0,928 | 1,456 | 0,190 |
|  | dominant | AAvsAG+GG | 1,033 | 0,880 | 1,214 | 0,691 |  |  | dominant | AAvsAG+GG | 1,040 | 0,871 | 1,241 | 0,668 |
|  | recessive | AA+AGvsGG | 1,050 | 0,881 | 1,252 | 0,586 |  |  | recessive | AA+AGvsGG | 1,172 | 0,967 | 1,421 | 0,106 |
| rs10819699 | additive | AGvsGG | 1,056 | 0,896 | 1,243 | 0,517 |  | rs10819699 | additive | AGvsGG | 0,986 | 0,824 | 1,179 | 0,875 |
|  |  | AAvsGG | 1,000 | 0,804 | 1,243 | 0,997 |  |  |  | AAvsGG | 0,952 | 0,749 | 1,210 | 0,689 |
|  | dominant | GGvsGA+AA | 1,040 | 0,892 | 1,214 | 0,614 |  |  | dominant | GGvsGA+AA | 0,977 | 0,825 | 1,157 | 0,786 |
|  | recessive | GG+GAvsAA | 0,969 | 0,796 | 1,179 | 0,752 |  |  | recessive | GG+GAvsAA | 0,960 | 0,774 | 1,192 | 0,712 |

The [95%] confidence interval is indicated for all different statistical models (additive, dominant, recessive) for all investigated SNPs.
