# Supplementary material for: Common polymorphisms within the NR4A3 locus, encoding the orphan nuclear receptor Nor-1, are associated with enhanced β-cell function in non-diabetic subjects
Source: BMC Med Genet. 2009 Aug 14;10:77. doi: 10.1186/1471-2350-10-77 (PMC2741445; doi:10.1186/1471-2350-10-77)
Supplement: Additional file 3 — Distribution of NR4A3 SNP minor allele frequencies according to glucose tolerance status in the TÜF/TULIP (N = 1495) and the METSIM (N = 6147) cohort. Minor allele frequencies for each investigated NR4A3 SNP are presented for study participants (TUEF-TULIP/METSIM trial) with normal glucose tolerance (NGT), impaired fasting glucose and/or impaired glucose tolerance (IFG/IGT) and with manifest diabetes (METSIM participants only). p1 – NGT vs. IFG/IGT in TUEF-TULIP/METSIM(χ2-test); p2 – NGTvs.IFG/IGTvs.DIABETESinMETSIM(χ2-test). SNPs screened in TUEF/TULIP only and not replicated in METSIM are marked with an asterisk. [file 1471-2350-10-77-S3.doc]

**Additional file 3** – Distribution of *NR4A3* SNP minor allele frequencies according to glucose tolerance status in the TÜF/TULIP (N=1495) and the METSIM (N=6147) cohort.

| SNP | Glucose Tolerance Status | | |  |  |
| --- | --- | --- | --- | --- | --- |
|  | **NGT** | **IFG/IGT** | **DIABETES** | **p_1_** | **p_2_** |
| N (TUEF-TULIP/METSIM) | 1090/3590 | 405/1731 | -/898 |  |  |
| rs7047636* | 0.371/- | 0.369/- | - | 0.97/- | - |
| rs1526267 | 0.260/0.336 | 0.246/0.351 | -/0.338 | 0.55/0.33 | 0.62 |
| rs2416879* | 0.095/- | 0.112/- | - | 0.07/- | - |
| rs12686676 | 0.469/0.459 | 0.445/0.451 | -/0.466 | 0.43/0.55 | 0.75 |
| rs10819699 | 0.323/0.408 | 0.309/0.421 | -/0.410 | 0.66/0.35 | 0.66 |

Minor allele frequencies for each investigated *NR4A3* SNP are presented for study participants (TUEF-TULIP/METSIM trial) with normal glucose tolerance (NGT), impaired fasting glucose and/or impaired glucose tolerance (IFG/IGT) and with manifest diabetes (METSIM participants only). **p_1_** – NGT vs. IFG/IGT in TUEF-TULIP/METSIM (χ^2^-test); **p_2_** – NGT vs. IFG/IGT vs. DIABETES in METSIM (χ^2^-test). SNPs screened in TUEF/TULIP only and not replicated in METSIM are marked with an asterisk.
